# Misperceptions about out-partisans’ democratic values may erode democracy

**DOI:** 10.1038/s41598-022-19616-4

**Published:** 2022-09-29

**Authors:** Michael H. Pasek, Lee-Or Ankori-Karlinsky, Alex Levy-Vene, Samantha L. Moore-Berg

**Affiliations:** 1 Department of Psychology, University of Illinois Chicago, Chicago, IL 60607 USA; 2 Department of Political Science, Brown University, Providence, RI 02912 USA; 3 Department of Political Science, University of Bath, Bath, BA2 7AY UK; 4 Annenberg School for Communication, University of Pennsylvania, Philadelphia, PA 19104 USA

**Keywords:** Psychology, Human behaviour

## Abstract

Two studies (one preregistered) of Americans (*N* = 2200) drawn from a nationally representative panel show that both Democrats and Republicans personally value core democratic characteristics, such as free and fair elections, but severely underestimate opposing party members’ support for those same characteristics. Democrats estimate that the average Democrat values democratic characteristics 56% (in Study 1) and 77% (in Study 2) more than the average Republican. In a mirror image, Republicans estimate that the average Republican values democratic characteristics 82% (in Study 1) and 88% (in Study 2) more than the average Democrat. In turn, the tendency to believe that political ingroup members value democratic characteristics more than political outgroup members is associated with support for anti-democratic practices, especially among Republicans. Results suggest biased and inaccurate intergroup perceptions may contribute to democratic erosion in the United States.

*"The Republicans have to get tougher. You're not going to have a Republican Party if you don't get tougher…When you catch somebody in a fraud, you're allowed to go by very different rules.”*

President Donald J. Trump, January 6, 2021.

Experts warn that American democracy is eroding and there is widespread evidence that core democratic practices, such as free and fair elections, are under threat^1–3^. While a majority of Americans now believe that American democracy is in crisis^4^, public opinion polls consistently show that, on an individual level, both Democrats and Republicans highly value democratic principles^5^. This reality illuminates an apparent paradox: if Americans across the political spectrum personally believe in the importance of core democratic characteristics, why are these principles being violated with increasing frequency?

To answer this question, we integrate recent insights from psychology and political science to test whether psychological biases stemming from social identity processes and political sectarianism may be associated with democratic norm erosion. Specifically, we suggest that Americans may form biased perceptions of how much members of their own and opposing parties value democratic characteristics. In turn, we suggest that such a tendency to underestimate out-partisan support for democratic principles, by undermining democratic norm strength, may be associated with a willingness to justify anti-democratic practices that serve partisan aims.

In its simplest form, democracy can be defined as a political system in which power is vested in the people. Influential accounts of democracy identify two important dimensions: (1) the possibility of public contestation, and (2) broader rights to participate in elections and serve as elected officials^6^. But for democracy to work in practice, there must be a balance between these two dimensions, so that the intensity of contestation does not compromise rights. To achieve that, citizens must abide by a set of “rules and procedures for waging and managing conflict by institutionalized means”^7^. These rules entail core democratic characteristics, including free elections, protections of basic rights, and restraints on the abuse of power^2,5,8^. Partisan adherence to these rules is buoyed by a logic of mutual deterrence, with each side recognizing that any violation committed by their side can be used to justify the same or worse actions by competing parties^9^.

Importantly, the rules that govern democratic practice extend beyond formal constitutional mechanisms and are bolstered by informal social norms. Social norms are shared understandings of values and behaviors that are appropriate and/or common in any given context. By promoting adherence to common behaviors and values, social norms enable humans to thrive in collectives^10^. Democratic norms can therefore be conceptualized as a particular set of social norms that guide the parameters of appropriate behavior in democratic societies. When understood as social norms, it becomes clear that democratic norms are not the aggregate individually held beliefs about the importance of democratic characteristics. Rather, they reflect mutual expectations about the degree to which others who participate in the same democratic arena value and abide by a shared set of rules that govern democratic practice^11^.

Democratic norm strength requires partisans to not only adhere to the same set of rules, but also to expect conformity from those belonging to the opposite party. If voters come to believe that pro-democratic views and behaviors are not normative among opposing party members, and that opposing party members fail to stop democratic abuse by their elected representatives, they may justify their own party engaging in undemocratic behaviors. Why? Because such actions may appear to be the only way to preemptively protect their interests against imminent violation. Indeed, one of the essential functions of democratic norms is to ensure the ability for those who are currently out of power to fairly contest to regain it. To the degree that people come to believe that their opponents have no regard for such norms, the prospect of political loss becomes untenable and anti-democratic behaviors may come to be perceived as justified, even necessary.

Despite strong evidence that both Democrats and Republicans do value democratic principles, psychological biases—likely fueled by elite behavior—may lead members of both parties to believe that pro-democratic views are non-normative among political opponents. This theorizing is grounded in research on political sectarianism^12,13^, which documents how American political parties have morphed into mega identities that represent more than mere ideological coalitions^14^. As a result, political parties, like other forms of social group membership, foster an “us” vs. “them” way of thinking that gives rise to numerous forms of intergroup bias^15^. In this work, we focus on one form of bias—the tendency to misperceive the motives and beliefs of out-partisans.

A growing body of research highlights how this “us” vs. “them” psychology can lead partisans to attribute negative motives and beliefs to opposing party members and to exaggerate the size of ideological differences. For example, Democrats and Republicans in the United States wrongly assume that their ingroup members are motivated by love whereas outgroup members are motivated by hate. In turn, this motive asymmetry drives intergroup conflict^16^. Similarly, Democrats and Republicans severely overestimate the extent to which members of their opposing party dislike and even dehumanize members of their own party^17,18^. In turn, this misestimation drives reciprocal dislike and dehumaniztion, exacerbates conflict, and even leads people to endorse anti-democratic behaviors^17,18^. These biases extend to issue-based polarization. Studies repeatedly show that Democrats and Republicans severely overestimate the size of their ideological differences on important political issues^18,19^, which can fuel political obstructionism^19^. Notably, these biases generlaize to other partisan conflicts^20^ and can fuel partisan violence^21^. This growing body of work suggests that a generalizable pattern whereby exagerated negative perceptions about what outgroup members believe and how they behave are associated with the tendency to justify one’s own (or one’s group’s) negative attitudes and behaviors. Such a tendency may also help to explain democratic norm errosion.

Just as psychological biases lead partisans to attribute negative motives and beliefs to their opponents, we reasoned that similar biases may lead Democrats and Republicans to (incorrectly) believe that members of their own party value democratic principles more than do members of their opposing party. Such biases might be quite pronounced given recent high-profile accusations by political elites in the United States about their opponents’ anti-democratic behavior. For example, widespread misinformation about voter fraud and the 2020 election being stolen may lead Republicans to believe Democrats have little regard for democratic principles. And to the contrary, Republican attempts to overturn legitimate election results and suppress voter turnout may lead Democrats to doubt Republicans’ commitment to democracy. Because we hypothesized such biases stem at least in part from social identity processes—through which individuals are motivated to attribute more positive traits to ingroup members—we also reasoned that these biases would be more pronounced among Democrats and Republicans who more strongly identified with their party. Although the focus of our research is on Democratic and Republican citizens’ perceptions of each other, we also posited that these same psychological biases apply to perceptions of political elites, whose anti-democratic behaviors have an outsized influence on democratic norm erosion and shape public opinion more broadly^22^.

We suggest that the tendency to believe that own-party members value democratic principles more than opposing party members may threaten democratic norm strength and practice. This misperception may upend the delicate balance of mutual deterrence, upholding democratic norms while generating incentives for preemptive anti-democratic actions. This could lead partisans to believe that the only way to compete for power is to engage in anti-democratic behaviors that they (wrongly) think outgroup members support. Put another way, even if Democrats and Republicans value democratic principles themselves, and believe that the violation of democratic principles is harmful for democracy, partisans who hold biased perceptions that such views are normative only among ingroup members may be more likely to support anti-democratic behaviors regardless. In so doing, similar to how pluralistic ignorance leads people to conform to views they disagree with^23^, individuals who hold negatively biased perceptions may behave in a manner that only reinforces out-partisans’ (inaccurate) perception that they do not value democratic principles. As a result, negatively biased perceptions might contribute to (and be fueled by) a downward spiral of democratic practice.

In the present research, we test our ideas in two studies—one preregistered—with self-identified Democrats and Republicans randomly sampled from a nationally representative panel maintained by YouGov. Here, we outline our core research questions, which themselves articulate our broader theoretical model. We first sought to replicate prior research documenting robust support for democratic principles among both Democrats and Republicans^5^. Next, we sought to test our novel prediction that, despite Democrats’ and Republicans’ support for democratic principles, members of both parties would wrongly believe that average members of their own party value democratic principles more than average members of their opposing party. In Study 1, we also explore whether these same biased perceptions apply to Democrats’ and Republicans’ perceptions of how much elites (i.e., members of Congress) from each party value democratic principles. Informed by social identity theory^15^, we also test whether biased perceptions are exaggerated among partisans who identify more strongly with their political party. Finally, we test whether individuals with more biased perceptions (i.e., those who more strongly believe that ingroup members value democratic principles more than outgroup members) are also more willing to endorse anti-democratic behaviors that advantage their own party. Across our research questions, we also explore differences by political party. Preregistration for Study 2, as well as well all data and code for both studies, are available at https://osf.io/h9dt8/?view_only=81b8d2ff931c4e60b8ea027e9f848daf.

## Results

### Democrats and republicans highly valued democratic characteristics

**Figure 1:**
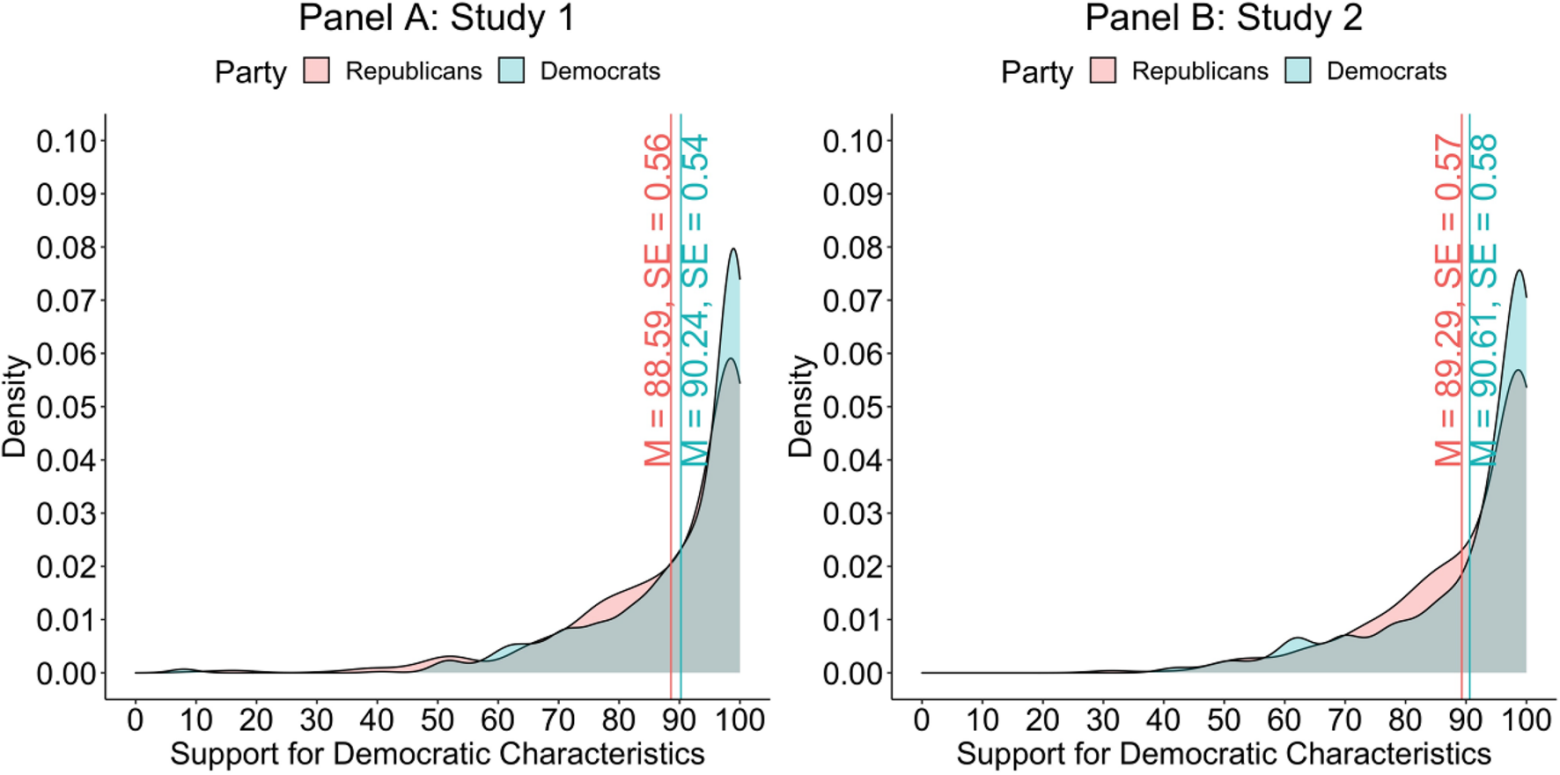
Density plots of respondents’ own ratings of democratic characteristics’ importance. Vertical bars denote means and standard errors for Republicans (red) and Democrats (blue).

Consistent with prior research^5^, in Study 1 and Study 2, members of both parties valued democratic characteristics, such as the importance of fraud-free elections. Notably, while Democrats (*M* = 90.24, *SE* = 0.54) valued democratic characteristics, on average, slightly more than Republicans (*M* = 88.59, *SE* = 0.56) in Study 1 (*t*[1221] = 2.12, *p* = 0.034, 95% CI[0.12, 3.17], η_p_^2^ = 0.004), no partisan difference between Democrats (*M* = 90.61, *SE* = 0.58) and Republicans (*M* = 89.29, *SE* = 0.57) emerged in Study 2 (*t*[976]  = 1.63, *p* = 0.104, 95% CI[− 0.27, 2.92], η_p_^2^ = 0.003). Results are displayed in Fig. 1.

### Democrats and republicans severely underestimated how much outgroup members valued democratic characteristics

**Figure 2:**
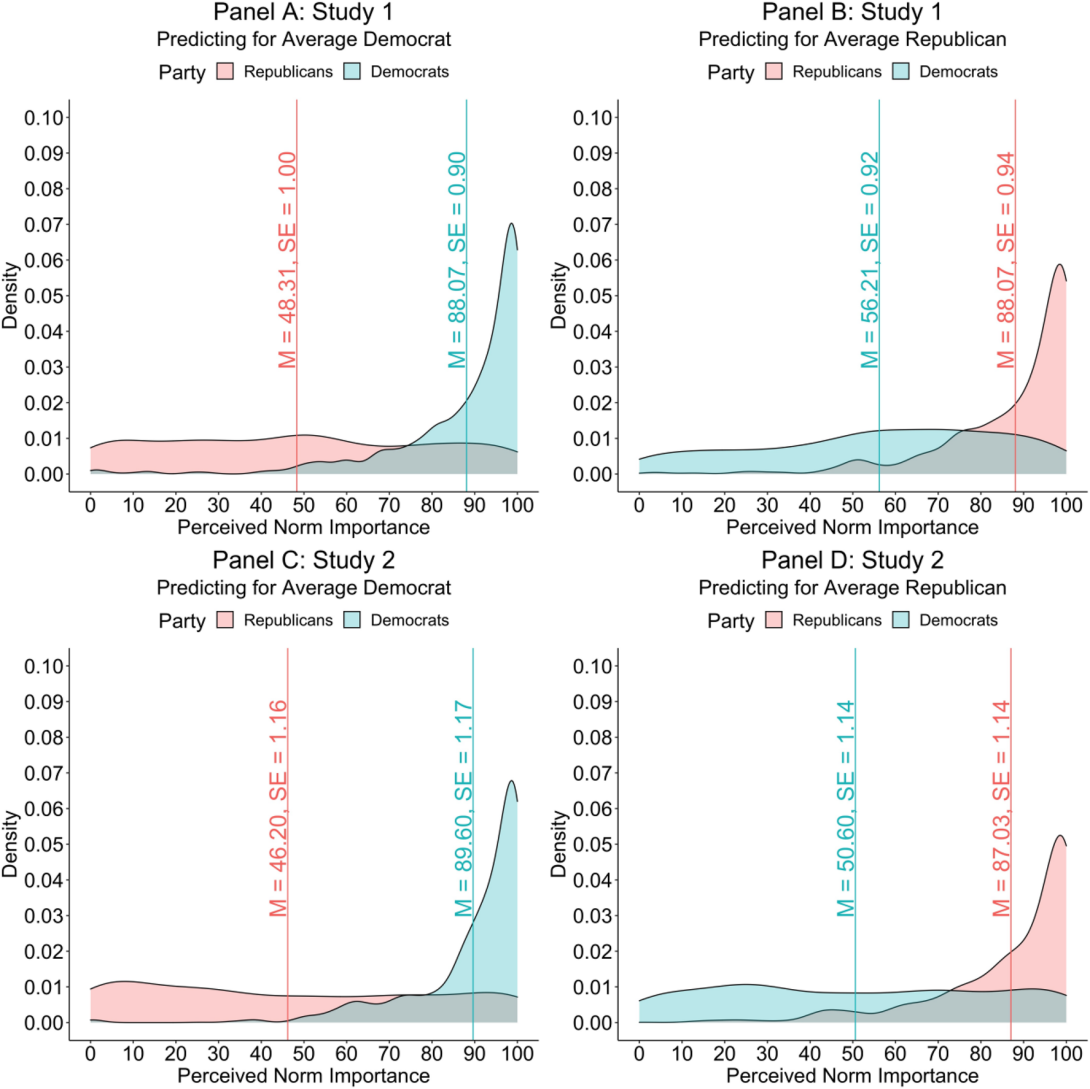
Density plots of partisans’ perceived norm importance. Panel (**A**) (Study 1) and Panel (**C**) (Study 2) demonstrates Republicans’ (red) and Democrats’ (blue) predictions about how much the average Democrat values democratic characteristics. Panel (**B**) (Study 1) and Panel (**D**) (Study 2) demonstrates Republicans’ (red) and Democrats’ (blue) predictions about how much the average Republican values democratic characteristics. Vertical bars denote means and standard errors for Republicans (red) and Democrat (blue) respondents.

Consistent with our theorizing, Democrats and Republicans in both studies believed that own-party members valued democratic characteristics more than opposing-party members. Democrats thought their ingroup would value the characteristics 31.86 point more (on a 0-to-100-point scale) in Study 1 (*t*[1221] = 24.59, *p* < 0.001, 95% CI[29.29, 34.43], η_p_^2^ = 0.326) and 39.00 points more in Study 2 (*t*[975] = 22.99, *p* < 0.001, 95% CI[35.67, 42.33], η_p_^2^ = 0.352) than their outgroup. In relative terms, democrats thought that own party members valued democratic characterizes 56% (in Study 1) and 77% (in Study 2) more than out-party members. Similarly, Republicans predicted their ingroup (compared to their outgroup) would value the characteristics 39.76 points more in Study 1 (*t*[1221] = 28.33, *p* < 0.001, 95% CI[37.01, 42.52], η_p_^2^ = 0.397) and 40.83 points more in Study 2 (*t*[975] = 24.21, *p* < 0.001, 95% CI[37.52, 44.14], η_p_^2^ = 0.375). In relative terms, Republicans thought that own party members valued democratic characterizes 82% (in Study 1) and 88% (in Study 2) more than out-party members. These results are displayed in Fig. 2, which demonstrates the minimal overlap between Democrats and Republicans’ perceptions.

Framed another way, Democrats’ and Republicans’ predictions about which group valued the characteristics more diverged by 71.62 points in Study 1 (*t*[1221] = 36.20, *p* < 0.001, 95% CI[67.74, 75.50], η_p_^2^ = 0.518) and 79.83 points in Study 2 (*t*[975] = 33.38, *p* < 0.001, 95% CI[75.14, 84.52], η_p_^2^ = 0.533). In both studies, this gap was significantly wider among stronger partisans (Study 1: *b* = 38.96, *t*[973] = 7.56, *p* < 0.001, 95% CI [28.85, 49.07], η_p_^2^ = 0.056; Study 2: *b* = 41.11, *t*[973] = 8.06, *p* < 0.001, 95% CI [31.10, 51.12], η_p_^2^ = 0.063). Among strong partisans, predictions made by Democrats and Republicans differed by 83.46 points in Study 1 (*t*[1219] = 36.56, *p* < 0.001, 95% CI [78.99, 87.94], η_p_^2^ = 0.523) and 91.88 points in Study 2 (*t*[973] = 33.24, *p* < 0.001, 95% CI [86.46, 97.31], η_p_^2^ = 0.532). Among weaker partisans, predictions differed by 49.66 points in Study 1 (*t*[973] = 11.58, *p* < 0.001, 95% CI [41.24, 58.08], η_p_^2^ = 0.121) and 50.78 points in Study 2 (*t*[973] = 11.85, *p* < 0.001, 95% CI [42.36, 59.19], η_p_^2^ = 0.126).

**Figure 3:**
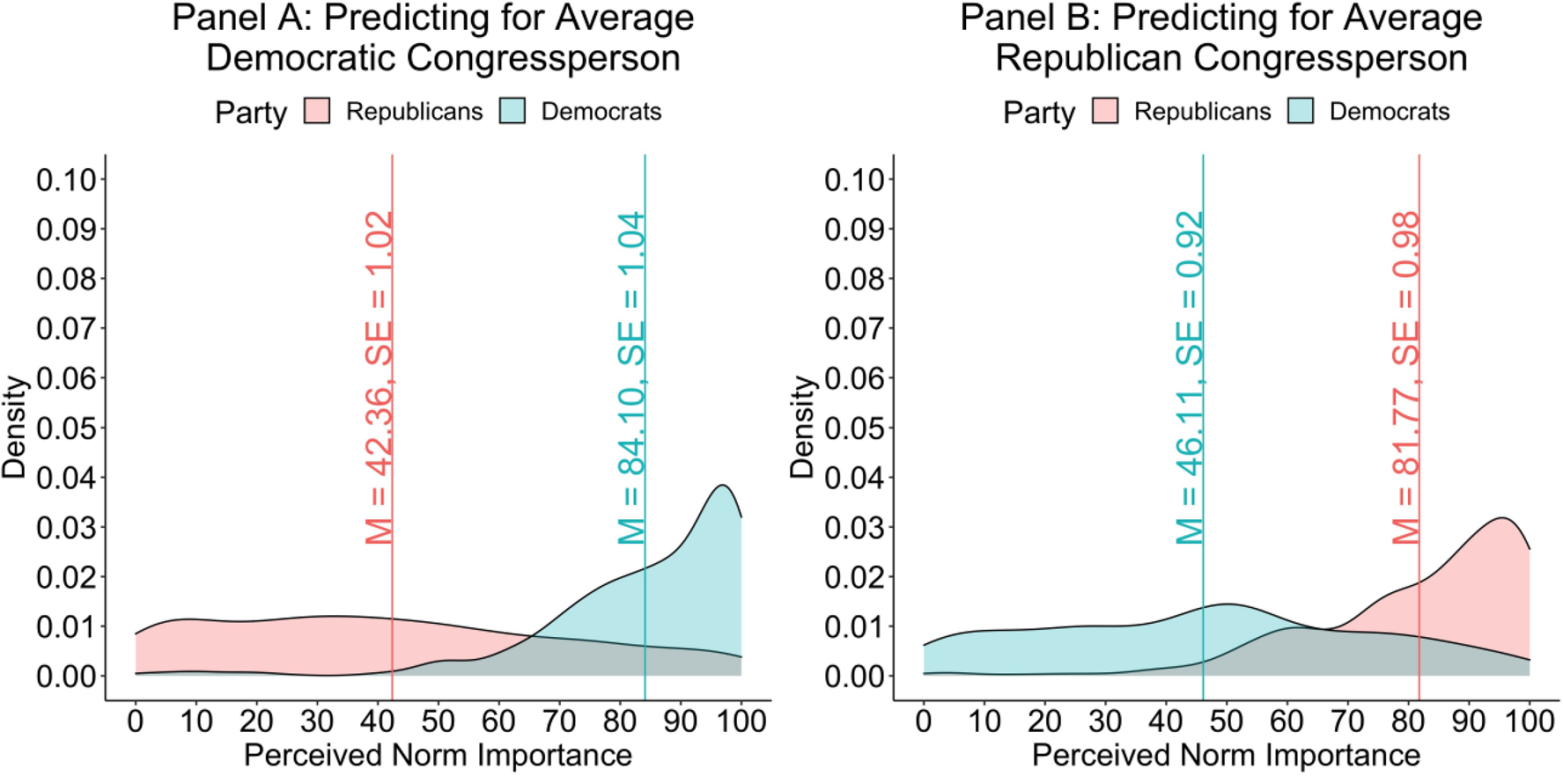
Density plots depicting how much partisans think Democratic (Panel **A**) and Republican (Panel **B**) congresspersons value democratic characteristics. Republicans’ perceptions are plotted in red, as are means and standard errors. Democrats’ perceptions are plotted in blue, as are means and standard errors.

In addition to our core questions, which pertain to perceptions about everyday citizens’ values, we also asked Study 1 participants (in an exploratory follow-up survey conducted two months later) to predict the degree to which the average Democratic and Republican congressperson valued the same principles. Results closely mirrored predictions made about the average Democrat and Republican. Democrats predicted the average Democratic congressperson (*M* = 84.10, *SE* = 1.04) would value the characteristics 37.99 points higher than the average Republican congressperson (*M* = 46.12, *SE* = 1.00; *t*[1024] = 24.76, *p* < 0.001, 95% CI[34.98, 41.00], η_p_^2^ = 0.374). Conversely, Republicans predicted the average Republican congressperson (*M* = 81.77, *SE* = 0.98) would value the characteristics 40.03 points higher than the average Democratic congressperson (*M* = 41.74, *SE* = 1.02; *t*[1024] = 26.57, *p* < 0.001, 95% CI[37.07, 42.98], η_p_^2^ = 0.401). In relative terms, Democrats and Republicans respectively predicted that the average congressperson from their own party would value democratic characteristics 82% and 93% more than the average congressperson from their opposing party member. Results are displayed in Fig. 3. As with perceptions about everyday citizens, the 78 point gap between each parties’ predictions (*t*[1024] = 36.28, *p* < 0.001, 95% CI[73.80, 82.23], η_p_^2^ = 0.562) was significantly wider among strong (as opposed to weak) partisans (*b* = 33.76, *t*[1022] = 7.18, *p* < 0.001, 95% CI[24.45, 42.99], η_p_^2^ = 0.048). While overall results here mirrored those reported above, comparison of Figs. 2 and 3 shows that partisans viewed support for democratic characteristics as slightly weaker among elites (compared to average partisans). This was true even when rating members of their party.

### Americans (especially republicans) with more biased intergroup perceptions were more approving of anti-democratic practices

**Figure 4:**
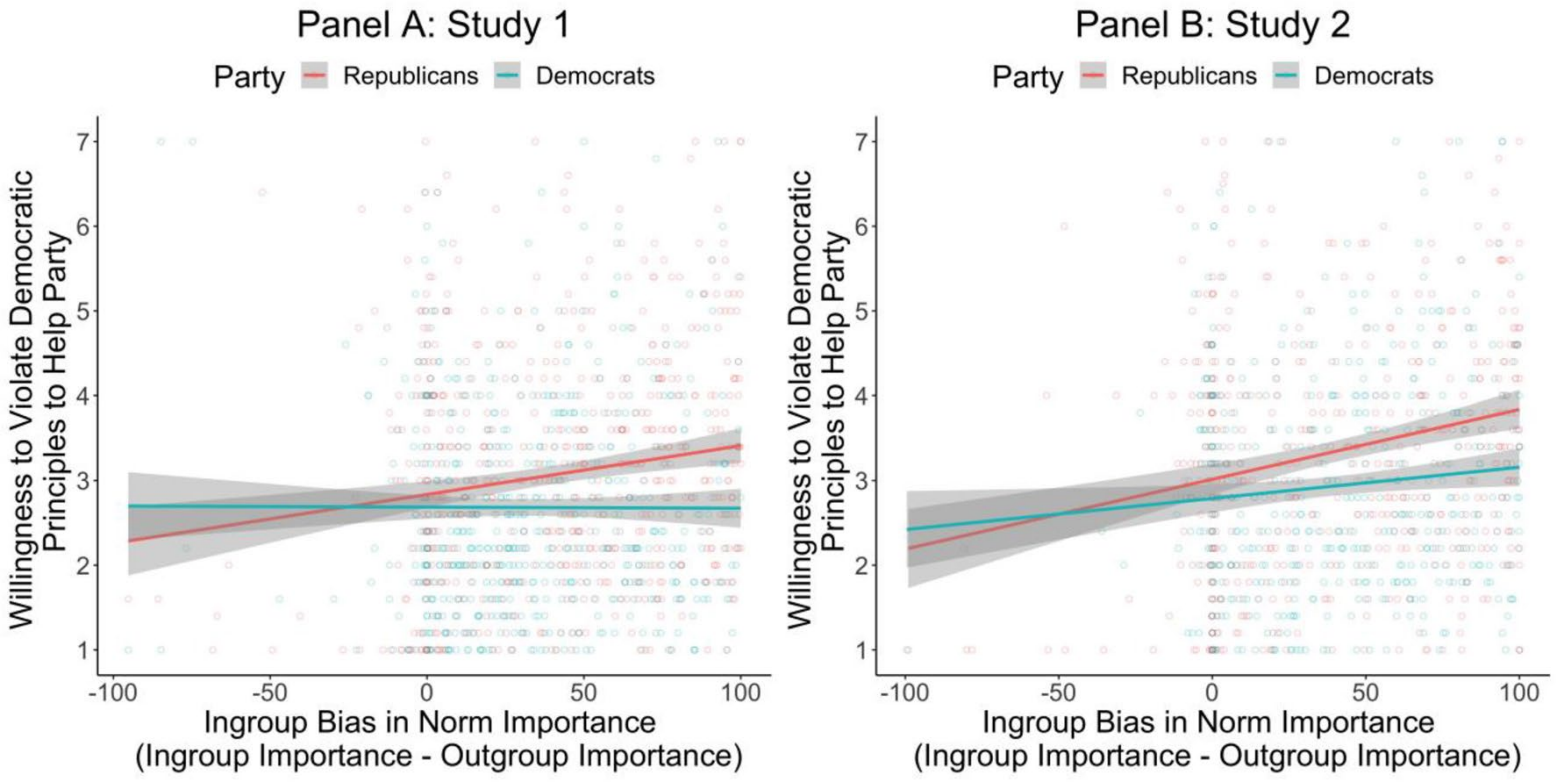
Relation between ingroup bias in perceived norm importance and willingness to violate democratic norms, with 95% Cis. Scatterplot shows underlying distribution for all respondents’ data.

We theorized that Americans with more biased intergroup perceptions would also be more approving of anti-democratic practices. As expected, in both studies, individuals who more strongly believed that the average ingroup member valued democratic characteristics more than the average outgroup member were also more willing to subvert democratic principles, in practice, to help their party (Study 1: *b* = 0.37, *t*[1221] = 3.49, *p* = 0.001, 95% CI[0.16, 0.59], η_p_^2^ = 0.010; Study 2: *b* = 0.79, *t*[975] = 6.85, *p* < 0.001, 95% CI[0.57, 1.02], η_p_^2^ = 0.046). In Study 1, we found this relation held for Republicans (*b* = 0.58, *t*[1219] = 4.03, *p* < 0.001 , 95% CI[0.30, 0.86], η_p_^2^ = 0.013), but not for Democrats (*b* = − 0.01, *t*[1219] =  − 0.08, *p* = 0.934, 95% CI[− 0.33, 0.30], η_p_^2^ < 0.001); interaction (*b* = − 0.59, *t*(1219) =  − 2.73, *p* = 0.006, 95% CI[− 1.01, − 0.17], η_p_^2^ = 0.006). In Study 2, this relation held for both parties, but was stronger for Republicans (*b* = 0.83, *t*[973] = 5.31, *p* < 0.001, 95% CI[0.52, 1.13], η_p_^2^ = 0.028) than for Democrats (*b* = 0.37, *t*[973] = 2.16, *p* = 0.031, 95% CI[0.33, 0.70], η_p_^2^ = 0.005), interaction (*b* =  − 0.46, *t*[973] =  − 1.98, *p* = 0.048, 95% CI[− 0.91, − 0.00], η_p_^2^ = 0.004). Results are displayed in Fig. 4. Notably, a mini meta-analysis shows that the cross-study effect for Democrats was also non-significant, z = 1.46). As can also be seen in Fig. 4, on average, and in both studies, Republicans (*M*_Study 1_ = 3.07, *SE* = 0.05; *M*_Study 2_ = 3.35, *SE* = 0.06) were also more willing than Democrats (*M*_Study 1_ = 2.68, *SE* = 0.05; *M*_Study 2_ = 2.93, *SE* = 0.06) to subvert democratic norms in practice (Study 1: *t*[1221] =  − 5.14, *p* < 0.001, 95% CI[− 0.53, − 0.24], η_p_^2^ = 0.021; Study 2: *t*[975] =  − 4.81, *p* < 0.001, 95% CI[-0.59, − 0.25], η_p_^2^ = 0.022).

## Discussion

In two studies conducted with large samples of American partisans drawn (*N* = 2200) from a nationally representative panel, we found that both Democrats and Republicans highly valued democratic characteristics. While this finding, which is consistent with prior research^5^, offers room for optimism about the health of American democracy, it also raises an important question: given high levels of individual support for democratic principles, why do so many partisans engage in anti-democratic behavior and tolerate it by their elected representatives^24,25^? Our results provide one potential explanation for this paradox. By demonstrating that American partisans severely underestimate out-partisan support for bedrock democratic principles, we suggest that these intergroup misperceptions may independently jeopardize Americans’, and especially Republicans’, commitment to democracy.

A core contribution of this research is our theorizing about the nature of democratic norms, with implications for thinking about democratic norm erosion. Rather than conceptualize democratic norm strength as an aggregate of individually held beliefs about the importance of democratic principles, we join others who have recently begun conceptualizing democratic norms as the aggregate of individuals’ perceptions of how much others, and particularly out-partisans, value democratic principles^11^. If norm strength was measured using individually held opinions, our data would present a rather optimistic take. However, by focusing on perceptions—themselves a constitutive quality of social norms—our data demonstrate that democratic norm strength—and potentially practice—may be severely undermined by a partisan psychology of “us” vs. “them” thinking.

### Possible causal mechanisms

A natural question is *why* Democrats and Republicans underestimate each other’s support for Democratic principles. One explanation, grounded in social identity theory^15^, is that the same psychological processes that lead partisans to overestimate affective and ideological polarization influence intergroup perceptions more broadly^18–20^.

A second explanation may be that partisans infer beliefs and values from actions, even if these actions are not representative of all party members. For example, Democrats who witnessed some Republicans storm the Capitol in the aftermath of the 2020 election have good reason to suspect that anti-democratic values are normative among some Republican voters, who continue to vote for elected representatives who scholars warn consistently undermine democratic principles^2,3^. Such perceptions are likely only exacerbated by a hyperpolarized media landscape^26^, in which members of both parties are constantly exposed to narratives of anti-democratic behavior committed by out-partisans. In such a hyperpolarized landscape, even asymmetric anti-democratic attitudes could fuel symmetric misperceptions and kickstart a downward spiral.

A third related explanation is that anti-democratic behavior committed by partisan elites may trickle down to influence perceptions of partisan citizens^22^, whose votes ultimately give power to elites themselves. Our finding that partisans attribute more anti-democratic values to elites from their opposing (and even own) party provides some support for this explanation. However, given the asymmetric nature of some of our findings—which demonstrate a stronger relation between biased perceptions and support for anti-democratic behavior by Democrats and Republicans—this account does not, on its own, explain the symmetrical nature of observed biased misperceptions.

A fourth explanation is that political campaigns and political media benefit from exaggerating the threat posed by out-partisans. Political campaigns (and aligned media organizations) must mobilize their supporters to donate funds, get out the vote, attend rallies, and call legislators—all costly activities. Increasingly, they must do so on a national level. More even-handed political media is incentivized to broadcast conflictual, threat-laden content to attract viewers. This creates a kind of escalation. Since voters may become desensitized to threats, the threshold for activation may climb higher and higher, requiring a greater “dose” of outsider threat for mobilization and attention. Future research should aim to test these four possible causal mechanisms.

The present research also contributes to an emerging interdisciplinary literature on the role of inaccurate intergroup perceptions in democratic decline. Notably, prior research demonstrates that correcting inaccurate intergroup perceptions about affective polarization can reduce toxic polarization and even partisan violence^21^. However, such corrections—which to date have focused on perceptions about affective and ideological polarization—have yet to counteract anti-democratic attitudes^27^. It is plausible that these interventions may fall short because they have not directly targeted perceptions about democratic values. Our results suggest that democratic erosion may be caused, at least in part, by a misguided fear that out-partisans do not share the same democratic values and, as a result, will not abide by the rules of the game. Thus, one potentially promising intervention strategy would be to directly assure Americans of their opposing party’s robust support for shared democratic principles. We are aware of two not yet published attempts to do so, both of which followed a preprint of an earlier version of this work. One experiment yielded significant but only small effects^28^, suggesting that, given a hyperpartisan media, anti-democratic behavior by partisan elites, and recent examples of citizens storming the capitol at the behest of their party leader, it may be difficult for an informational intervention to effectively counteract the many forces fueling partisan misperceptions. However, a separate set of studies reported in a recent preprint not only conceptually replicates our general finding that Democrats and Republicans who most fear that out-partisans will subvert democracy are themselves more willing to subvert democratic principles, but also extends this finding by experimentally showing that an intervention correcting inaccurate misperceptions about out-partisans’ commitment to democracy promotes adherence to democratic norms^29^. Future research is needed to identify what makes some informational interventions more effective than others.

### Alternative explanations for our findings

While our research, and emerging complementary work^29^, suggests that biased intergroup perceptions may help to explain the seeming paradox between Americans’ support for democratic principles and the simultaneous erosion of democratic practice, it is important to consider alternative explanations and the potential for bidirectional processes. We review four non-exhaustive possibilities, each of which deserves independent inquiry.

One alternative explanation could be that negative partisanship^30^ and affective polarization^12^ are so strong that they override Americans’ otherwise pro-democratic attitudes. Thus, it is possible that the cross-sectional relation observed in the present research—between misperceptions and anti-democratic attitudes—emerges from a third variable. While this may explain part of our observations, the asymmetrical relation between misperceptions and anti-democratic views, with stronger relations among Republicans than Democratic, suggests that partisan animosity on its own does not offer a full mechanistic explanation. Relatedly, one might ask what causes the asymmetry in the first place? One potential explanation could be that our studies were conducted shortly after the Democrats took control of the presidency and congress, making the stakes much larger for Republicans, who were ousted from political power. This may help to explain why the relation between biased perceptions and support for anti-democratic behaviors was stronger for Republicans in both studies. Nonetheless, given the cross-sectional nature of our data, we are not able to fully rule out third-variable effects or to confidently identify the source of observed asymmetry. Future experimental and/or longitudinal research is needed to address these questions.

A second alternative explanation for this incongruence may be that partisans have different views about what actions constitute a threat to democratic principles. For example, voter suppression laws are often framed as protecting election integrity. This suggests that proponents of anti-democratic policies may recognize democratic principles’ widespread popularity and market anti-democratic proposals in pro-democratic language. This may help to explain why many Republican voters and elites defended efforts to overturn the 2020 presidential election as a way to protect democratic processes. Relatedly, racial animus underlying support for Donald Trump^31^, as well as racialized conceptions of who is American^32^, may foster alternative views about how to defend democracy. As a result, anti-democratic behaviors that disproportionately disenfranchise racial minorities, such as voter suppression^33^, can be perversely seen as either protecting American democracy or hurting it, depending on the accuracy of one’s preconceptions. Future research should explore these possibilities.

A third alternative explanation is that pro-democracy views may not be as deeply held as many Americans self-report. For example, it is possible that Democrats or Republicans overestimate the extent to which they and their own party value democratic norms (rather than underestimate how much out-partisans value democratic norms). This could result from social desirability bias^34^ or stem from a gap between the values individuals hold in abstract and how they put these values into practice. For example, partisans may view democratic principles that they support as fungible and worth sacrificing for competing values, such as power. And for many segments of the population (e.g., White Christians), for whom increasing diversity threatens political power, there may be a newfound tension between pro-democratic views and pro-group views^35–38^. This broader context may also help to explain why a willingness to subvert ostensibly held democratic values was higher among Republicans. This question remains ripe for future research.

Fourth, it is also possible that the relation between democratic norm adherence and (mis)perceptions about out-partisans commitment to democracy is bidirectional. That is, just as partisans may be more willing to subvert democratic principles that they hold important if they believe that out-partisans won’t play by the rules, partisans may also seek to downplay out-partisans’ commitment to democracy to justify their own (or their group’s) anti-democratic behavior. Thus, misperceptions may help individuals maintain a positive view of their group and/or serve as a moral license to continue undemocratic behavior.

Democratic strength requires that citizens adhere to—and remove from office elites who violate—bedrock principles of democracy. The maintenance of strong democratic norms ensures that parties have a fair chance to compete for power and that minority rights are protected. While it is important that citizens value democratic principles, doing so may not be enough to ensure that they also uphold such principles in practice. Democratic norm strength requires that partisans from all parties not only personally value democratic principles, but also believe that members of their own and opposing parties do the same. This mutual perception, which itself can be conceptualized as a metric of democratic norm strength, is easily jeopardized in a sectarian political climate marked by a hyper-partisan psychology. Democratic health requires attention to this psychology.

## Method

### Participants

Respondents for both studies were sampled from YouGov, which maintains a large opt-in panel of Americans, from which we drew nationally representative samples using YouGov’s Active Sampling methodology. Respondents were compensated directly by YouGov for participating in this research. See Table 1 for sample information. Ninety-two percent of Study 1 participants completed the follow-up survey in which perceptions about congresspersons were assessed. Independents were excluded in both studies (as preregistered in Study 2). Respondents were strong (Study 1 = 72%; Study 2 = 71%) or not very strong (Study 1 = 28%; Study 2 = 29%) partisans. Data provided by YouGov only included participants who passed an embedded attention check.

**Table 1:** Demographics.

	Study 1			Study 2		
	Total sample *N* = 1223	Democrats *n* = 623	Republicans *n* = 600	Total sample *N* = 977	Democrats *n* = 488	Republicans *n* = 489
Mean Age (SD)	53.74 (16.21)	51.52 (16.28)	56.04 (15.82)	52.41 (16.34)	49.64 (16.64)	51.57 (15.57)
Gender	45% Male, 55% Female	39% Male, 61% Female	51% Male, 49% Female	41% Male, 59% Female	36% Male, 64% Female	47% Male, 53% Female
Race	79% White, 9% Black, 7% Hispanic, 2% Asian, < 1% Native American, < 1% Mixed, 1% other	70% White, 16% Black, 8% Hispanic, 3% Asian, < 1% Native American, 1% Mixed, < 1% other	88% White, 2% Black, 6% Hispanic, 2% Asian, < 1% Native American, 0% Mixed, 2% other	77% White, 9% Black, 7% Hispanic, 2% Asian, < 1% Native American, 2% Mixed, 2% other	69% White, 17% Black, 7% Hispanic, 3% Asian, < 1% Native American, 3% Mixed, 1% other	85% White, 2% Black, 7% Hispanic, 1% Asian, < 1% Native American, 1% Mixed, 2% other

### Materials and procedure

Respondents completed online surveys in March (Study 1), June (Study 1 follow-up), and June and July (Study 2) of 2021. Both studies were completed as part of separate larger surveys, which were conducted with IRB approval from the University of Pennsylvania (#823959). All methods were carried out in accordance with relevant guidelines and regulations, and informed consent was obtained from all subjects. Relevant measures are described below. Additional materials and corresponding analyses can be found in Supplementary Information.

#### Party identification and partisan strength

Respondents indicated whether they identified as Democrats, Republicans, Independent, other, or not sure. Only those who indicated Democrat or Republican were included. A second question asked whether respondents identified as strong or weak members of their respective political party. This question also allowed respondents to indicate whether they leaned toward one party or the other, although, after removing independents, all partisans identified as either strong or not very strong partisans.

#### Actual and perceived importance of democratic characteristics

Democratic characteristics were selected from Bright Line Watch^5^ to represent four key principles. (1) “Elections are conducted, ballots counted, and winners determined without pervasive fraud or manipulation.” (2) “All adult citizens enjoy the same legal and political rights.” (3) “Government agencies are not used to monitor, attack, or punish political opponents.” And (4) “Law enforcement investigations of public officials or their associates are free from political influence or interference.” Participants rated how important they found these characteristics on a sliding scale (0 = not at all important to 100 = extremely important). Items were averaged (α_Study 1_ = 0.82; α_Study 2_ = 0.80). Participants also rated how important the average Democrat (α_Study 1_ = 0.96; α_Study 2_ = 0.96) and Republican (α_Study 1_ = 0.93; α_Study 2_ = 0.95) would find these characteristics. Study 1 participants, in a follow-up, indicated how important the average Democratic (α = 0.94) and Republican (α = 0.91) congressperson would find these characteristics (see Supporting Information).

#### Willingness to violate democratic norms to help one’s party

Five items (Republican items in brackets) assessed support for anti-democratic practices^18^. “I think Democrats [Republicans] should do everything in their power within the law to make it as difficult as possible for Republicans [Democrats] to run the government effectively.” “I think Democrats [Republicans] should do everything they can to hurt the Republican [Democratic] Party, even if it is at the short-term expense of the country.” “Democrats [Republicans] should redraw districts to maximize their potential to win more seats in federal elections, even if it may be technically illegal.” “Since Democrats gained [if Republicans gain] control of all branches of government in 2020 [2024], they should use the Federal Communications Commission to heavily restrict or shut down Fox News [MSNBC] to stop the spread of fake news.” And “It’s OK to sacrifice US economic prosperity in the short-term in order to hurt Republicans’ [Democrats’] chances in future elections.” Items (7-point strongly disagree to strongly agree scale) were averaged (α_Study 1_ = 0.76; α_Study 2_ = 0.77).

### Analytic strategy

Results were analyzed using weighted regressions. Weights supplied by YouGov were applied to provide population-level estimates. Respondents were weighted to their respective parties using propensity scores including age, gender, race/ethnicity, years of education, and region. Weights were post-stratified based on these criteria as well as 2016 and 2020 presidential vote choice.

For main analyses, we contrast coded political party (− 0.5 = Republican, 0.5 = Democrat). Marginal means and simple slopes were calculated using dummy codes. For analyses involving party strength moderation, strong partisans were coded 0.5 and weak partisans were coded − 0.5. Simple slopes were again analyzed using dummy codes.

To investigate associations between biased meta-perceptions and support for anti-democratic practices, we subtracted outgroup norm predictions from ingroup norm predictions. To ease parameter interpretation, we divided this score by 100. Removing observations >|3| studentized deleted residuals did not meaningfully influence results.

Given the asymmetrical relation between biased perceptions and one’s own willingness to violate democratic norms, which was stronger for Republicans than Democrats, we calculated a cross-study effect by conducting a mini meta-analysis. Following recommended practices, we converted observed *t*-values to correlations, which were weighted and pooled to calculate a cross-study z-score^39^.

## Supplementary Information

Supplementary Information.

## Data Availability

The datasets and code generated and analyzed for both studies are available in the Open Science Foundation repository at https://osf.io/h9dt8/?view_only=81b8d2ff931c4e60b8ea027e9f848daf.
