# Supplementary material for: Misperceptions about out-partisans’ democratic values may erode democracy
Source: Sci Rep. 2022 Sep 29;12:16284. doi: 10.1038/s41598-022-19616-4 (PMC9523060; doi:10.1038/s41598-022-19616-4)
Supplement: Supplementary file 1 — Supplementary Information. [file 41598_2022_19616_MOESM1_ESM.pdf]

Supplemental Materials

Misperceptions About Out-Partisans' Democratic Values May Erode Democracy

*Michael H. Pasek, Ph.D.[1], Lee-Or Ankori-Karlinsky[2],

Alex Levy-Vene[3], & Samantha L. Moore-Berg, Ph.D.[4]

[1]Department of Psychology, University of Illinois Chicago, Chicago, IL, 60607

[2]Department of Political Science, Brown University, Providence, RI, 02912

[3]Department of Political Science, University of Bath, UK, BA27AY

[4]Annenberg School for Communication, University of Pennsylvania, Philadelphia, PA, 19104

*Correspondence should be sent to Dr. Michael H. Pasek (mpasek@uic.edu).

Supplementary Measures and Analyses

In both studies, we included a measure intended to assess support for protest groups that engage in violence. We preregistered this measure and corresponding analyses in Study 2. As we report below, results were consistent with preregistered hypotheses. While we intended to make a measure that would assess similar beliefs for Democrats and Republicans, upon further reflection, we believe that our measure may tap different beliefs for each party. As described in more detail below, these measures assessed attitudes toward violent fictional protest groups that we explained were gathering at the Capitol. These groups were modelled after the real events of January 6, 2021. However, because we asked these questions after those events, in which supporters of President Trump actually subverted democratic principles and engaged in violence, the meaning of our measure may be substantively different for Democrats and Republicans. For Republicans, our measure (which based on real events) most likely taps support for violence to subvert democratic norms, whereas for Democrats, our measure (which was more hypothetical) may more aptly be assessing support for violence to defend against norm subversion. As such, we report this measure and accompanying results here as opposed to in the main text.

Specifically, both parties were told that, "As a response the events that unfolded at the capitol on January 6th, intended to disrupt the certification of President Biden, two groups of Americans have formed in Washington DC." "Inauguration Supporters" was described as "engag[ing] in… actions to prevent similar violence that occurred on January 6th from reoccurring in the future." "Inauguration Protestors" was described as "engag[ing] in…actions to…display they do not support the new presidential administration of President Biden." Democrats and Republicans were told that Inauguration Supporters and Inauguration Protestors, respectively, destroyed equipment belonging to the other group and used violence to stop members from the

other group. Participants were asked whether they would (1) consider joining, (2) support, and (3) identify with Inauguration Protestors (for Republicans) or Inauguration Supporters (for Democrats). Participants also indicated whether they (4) like that their respective group's willingness to use violence. Items (7-point strongly disagree to strongly agree scale) were averaged ($\alpha_{\text{Study 1}} = .87$, $M_{\text{Study 1}} = 3.10$, $SE_{\text{Study 1}} = .04$; $\alpha_{\text{Study 2}} = .86$, $M_{\text{Study 2}} = 3.19$, $SE_{\text{Study 2}} = 0.05$).

Consistent with hypotheses, individuals with more biased meta-perceptions were more supportive of party-aligned protest groups that engaged in violence (S1: $b = 0.63$, t[1121] = 5.01, $p < .001$, 95% CI[0.38, 0.87], $\eta_p^2 = .020$; S2: $b = 1.08$, t[975] = 8.35, $p < .001$, 95% CI[0.83, 1.34], $\eta_p^2 = .067$). Relations were not moderated by party (S1: $t[1219] = -1.30$, $p = .195$, $\eta_p^2 = .001$; S2: $t[973] = -0.74$, $p = .459$, $\eta_p^2 = .001$). No two-way partisan strength x ingroup bias or partisan strength by ingroup bias by party interactions emerged across outcomes or studies (*p*s > .05).

In S2, we additionally measured Democrats' and Republicans' support for Black Lives Matter and Blue Lives Matter. In our preregistration, we explicitly stated that these measures were exploratory. We asked members of both parties how much they would support peaceful and violent protestors from each group (0 = would not support at all, 100 = would fully support). We computed an alternative exploratory measure of support for partisan-aligned violence by using support for violent Black Lives Matter protestors for Democrats ($M = 20.27$, $SE = 1.22$) and support for violent Blue Lives Matter protestors for Republicans ($M = 2.94$, $SE = 0.07$). We then regressed this measure on our measure of biased meta-perceptions, again weighting respondents to their respective party. We did not find a significant relation ($t[975] = -1.51$, $p = .131$, $\eta_p^2 = .002$), nor was this relation moderated by party ($t[973] = -1.89$, $p = .059$, $\eta_p^2 = .004$).
